# Quality of life in patients with malignant pleural effusion treated with an indwelling pleural catheter in an emerging country

**DOI:** 10.1016/j.clinsp.2022.100063

**Published:** 2022-06-18

**Authors:** Leticia Leone Lauricella, Paula Duarte D'Ambrosio, Priscila Berenice da Costa, Marcia Cristina Augusto, Paulo Manuel Pêgo-Fernandes, Ricardo Mingarini Terra

**Affiliations:** a Instituto do Câncer do Estado de São Paulo (ICESP), Hospital das Clínicas, Faculdade de Medicina, Universidade de São Paulo (HCFMUSP), São Paulo, SP, Brazil; b Instituto do Coração, Hospital das Clínicas, Faculdade de Medicina, Universidade de São Paulo (HCFMUSP), São Paulo, SP, Brazil

**Keywords:** Brazilian public health system, Indwelling pleural catheter, Life expectancy, Malignant pleural effusion, Quality of life

## Abstract

**Background:**

The use of Indwelling Pleural Catheter (IPC) in the care of patients with Malignant Pleural Effusion (MPE) is well established, however studies involving public health systems of low and middle-income countries are still lacking. This study aimed to determine the effect of IPC on the respiratory symptoms and Quality of Life (QoL) of patients with MPE in the setting of a Brazilian public health system.

**Methods:**

From August 2015 to November 2019, patients with MPE underwent IPC placement and were prospectively followed. QoL and respiratory symptoms were assessed by the EORTC questionnaires (QLQ-30; LC13) and Visual Analogue Scale (VAS), respectively, at pre-treatment, 30 , and 60 days after IPC placement.

**Results:**

56 patients were enrolled with 57 catheters inserted. The mean age was 63 (23‒88) years, of which 17 (30%) were men and 39 (70%) were women. Breast 24 (42%) and lung 21 (37%) were the main primary neoplasms. Cellulitis was the most common complication and all patients recovered with appropriate antimicrobial therapy. QoL did not change significantly over time, however, the VAS showed a significant improvement in dyspnea (+1.2: -0.5; *p* = 0.001).

**Conclusion:**

IPC relieves respiratory symptoms without compromising the QoL, with a low complication rate. It represents a suitable option for patients with MPE and short LE in an emerging country.

## Highlights

• Indwelling pleural catheter represents a suitable option for patients with malignant pleural effusion and short life expectancy. It relieves respiratory symptoms without compromising the quality of life, and the complication rate is low, even in an emerging country, with a low socioeconomic and under-educated patient population. The rate of spontaneous pleurodesis was 45%. The analysis of the visual analog scale showed significant control of dyspnea (*p* = 0.001), but pain and quality of life did not change significantly.

## Introduction

Malignant Pleural Effusion (MPE) is a well-known sign of end-stage cancer that can reduce Life Expectancy (LE) with an average survival time of 3–12 months [1]. Dyspnea is not only the most common and often distressing symptom, found in over 50% of patients with MPE [2], but also the main cause of emergency care, leading to a negative impact on patients’ Quality of Life (QoL) [3,4]. Consequently, the main goal for the treatment of these patients is symptom palliation while minimizing the adverse events associated with invasive procedures.

The choice of therapy to manage MPE must consider the patient's clinical performance status and LE, prioritizing QoL improvement. Comparable results of the use of Indwelling Pleural Catheters (IPCs) versus pleurodesis in terms of symptom control and QoL [5] have increased the acceptance of IPC, which is now considered a suitable alternative to talc pleurodesis in many countries. Several studies have shown that IPC is a less invasive alternative, avoiding hospitalization and allowing domiciliary drainage and outpatient care [6,7]. Its proper use requires technical knowledge during catheter insertion and long-term care to prevent infectious complications. Catheter manipulation by the patients or their caregivers in the home environment is relatively simple but requires training and a good understanding of the device and disease symptoms. In this way, the use of IPC in the public health systems of low- and middle-income countries could be both a challenge and a limitation.

The adoption of IPC in developing nations has not been assessed thoroughly. This prospective study aimed to determine the benefits of IPC on the symptoms and QoL of patients with recurrent MPE in the context of the Brazilian public health system. Additionally, the authors aimed to evaluate the feasibility of IPC drainage.

## Material and methods

This prospective observational study was performed between August 2015 and November 2019 in a Brazilian public health system cancer center in the city of São Paulo. This study was approved by the institutional review board, and informed consent was obtained from all patients (CAPPesq HCFMUSP-882.696). Patients with MPE included in this study were recruited from an outpatient clinic. Patients were considered eligible if they had recurrent MPE (diagnosis confirmed by pleural fluid oncotic cytology or pleural biopsy), a Karnofsky Performance Status index greater than 50, and a life expectancy greater than 6-weeks. Patients younger than 18-years with current pleural infection, previous major pleural procedures (pleurodesis or decortication), hemorrhagic diathesis, or inability to understand the QoL questionnaires were excluded from the study.

### Data collection

Demographic data, including age, sex, type of malignancy, initial performance status, and smoking history, were collected at baseline. The size of the pleural effusion was defined semi-quantitatively by the investigators using a chest radiograph prior to IPC insertion as small (less than 1/3 of the hemithorax), medium (1/3–2/3 of the hemithorax), and massive (more than 2/3 of the hemithorax). The presence or absence of lung entrapment was also recorded. Patients completed the European Organization for Research and Treatment of Cancer QoL (EORTC QLQ-C30 Version 3.0) and Lung Cancer module (LC13) prior to catheter insertion (D0). To assess pain and dyspnea, the authors used a Visual Analog Scale (VAS). In all contacts with patients, the authors actively sought adverse events graded according to the Common Terminology Criteria for Adverse Events (CTCAE) of the National Cancer Institute v4.0 criteria [8].

### Questionnaires

In this study, the patients completed two Portuguese EORTC QoL questionnaires. The first self-report questionnaire assessed global QoL (EORTC-QLQ 30, version 3.0) [9]. The QLQ-30 comprises both multi-item and single-item scales, including five functional scales, three symptom scales, a global health status/QoL scale, and six single items. Each multi-item scale includes a different set of items, and no item occurs on more than one scale. For the functional scale and global health status, higher scores indicate better functioning. For symptom scales, higher scores represent a higher level of symptomatology.

The second self-report questionnaire assessed patients’ functions (QLQ-LC13 supplementary module). QLQ-LC13 includes questions assessing lung cancer-associated symptoms, treatment-related side effects, and pain medication. The lung cancer module incorporates a multi-item scale to assess dyspnea and a series of single items assessing pain, coughing, sore mouth, dysphagia, peripheral neuropathy, alopecia, and hemoptysis. The validity of these questionnaires has been confirmed by international studies on cancer-guided QoL instruments [10].

A VAS was used to evaluate pain and dyspnea, and it has ten numerical values, rated on a scale from 0 to 10, with each value corresponding to the intensity of breathlessness and painlessness [11].

### Surgical procedure

The procedure was performed in an operating room under sterile conditions with ultrasound guidance under local anesthesia. A PleurX catheter (E.-TAMUSSINO & CIA LTDA) was inserted using the Seldinger technique through the sixth or seventh intercostal space in the middle axillary line. [7] Chest radiography confirmed the catheter position.

### Drainage protocol

Upon discharge, the patients and caregivers were trained by the medical and nursing staff on catheter management. They were invited to watch a video showing the drainage process and receive printed material with instructions. They were advised to record the drainage volume and report any symptoms. A kit with ten drainage bottles was provided to the patient. They were instructed to drain up to 600 mL of pleural fluid using a sealed vacuum bottle 3-times a week. After the first month, the drainage frequency can be adapted according to the patient's average rate of fluid production or the development of respiratory symptoms. The IPC was removed once drainage had stopped for 2 consecutive weeks or had an output of <50 mL/day. In cases of major complications, the catheter can also be withdrawn. Spontaneous Pleurodesis (SP) was defined as the absence of further pleural intervention owing to MPE after IPC removal.

### Follow-up

The EORTC questionnaire and VAS were completed on D0, D30, and D60. Patients were examined in the outpatient clinic for evaluation at D30 and D60. At each visit, the site of insertion and catheter functioning were verified, and the patients underwent chest radiography. Additionally, the number of bottles used, and frequency of drainage was recorded. Complications related to the catheter were questioned during each follow-up period. After catheter removal, patients were followed up until death.

### Statistical analysis

The central tendency measures were calculated for continuous variables. Unless otherwise indicated, data are presented as the mean ± Standard Deviation (SD). In this study, Levene's test was used to assess the equality of variances. The EORTC QoL was scored according to the scoring manual [9]. QoL and VAS scores from baseline to 30-day follow-up were analyzed using a paired *t*-test after confirming the normality hypothesis. Separately, repeated measures in the analysis of variance were performed to compare QoL scores and VAS scores at baseline and 30-day and 60-day follow-ups. Fisher's exact test was used in the treatment of data regarding the association between initial performance status and mortality 30 and 60 days after IPC placement. Statistical significance was set at *p*<0.05. All data were analyzed using the SPSS software package (IBM Statistics) version 20.

## Results

Fifty-six patients underwent 57 IPC insertion procedures. One patient developed contralateral pleural effusion after treatment with IPC and underwent a new catheter insertion. The demographics of the cohort are presented in Table 1. Complications related to IPC were registered and graded according to the CTCAE grade system V4.0 [8], as detailed in Table 1. Cellulitis was the most common complication, and all patients recovered with appropriate antimicrobial therapy, without the need for catheter removal. Four patients had empyema, two required surgical intervention and removal of the IPC, one recovered with antibiotics, and one was referred to palliative care with no interventions other than comfort measures. No mortality was associated with catheter placement. The median patient consumption of bottles was 1.7 per week and 14.5 during the first 60 days after IPC. During the study period, 38 (67%) patients underwent IPC removal, with a mean of 47 days after catheter removal. However, eight patients required further pleural intervention. SP occurred in 17 of the 38 patients whose catheters were withdrawn, leading to an SP rate of 45%. At the end of the study period, 16 of the 56 patients enrolled had died (28%), and 14 of them still had the catheter in place.

**Table 1:** Baseline demographic data for 56 patients with malignant pleural effusion.

**Variables**		
Sex, n (%)	Female	39 (70)
	Male	17 (30)
Age, n (Max/Min)		63 (88/23)
Smoking history, n (%)	Smoking	1 (2)
	Ex-smoking	15 (27)
	Never	36 (64)
	Ignored	4 (7)
Etiology^a^, n (%)	Breast	24 (42)
	Lung	21 (36.8)
	Others	16 (21,2)
Karnofsky Scale (KPS), n (%)	51‒60	8 (14)
	61‒70	12 (22)
	71‒80	22 (39)
	81‒90	9 (16)
	91‒100	5 (9)
Lung pleural expansion, n (%)	> 90%	23 (41.1%)
	< 90%	33 (58.9%)
Size of Pleural effusion (before IPC), n (%)	Small (< 1/3 do hemithorax)	8 (14.3)
	Medium (1/3‒2/3 do hemithorax)	25 (44.6)
	Massive (> 2/3 do hemithorax)	23 (41.1)
Aspect of pleural effusion (RX ou CT), n (%)	Free	44 (79)
	Loculated	12 (21)
Number of pleural interventions, median (Max/Min)		1.68 (8/0)
Side of pleural effusion, n (%)	Right	38 (67.9)
	Left	14 (25.0)
	Bilateral	4 (7.1)
QT/RT^b^ previous, n (%)	Yes	26 (46.4)
	No	30 (53.6)

n, There were 56 patients enrolled in this study, with 57 IPC placed (one patient underwent a bilateral drainage). ^a^ As for the etiology, some patients with MPE had more than one type of cancer. ^b^ QT/RT, Quimio OR Radiation Therapy.

### Quality of life (QoL)

**Fig. 1:**
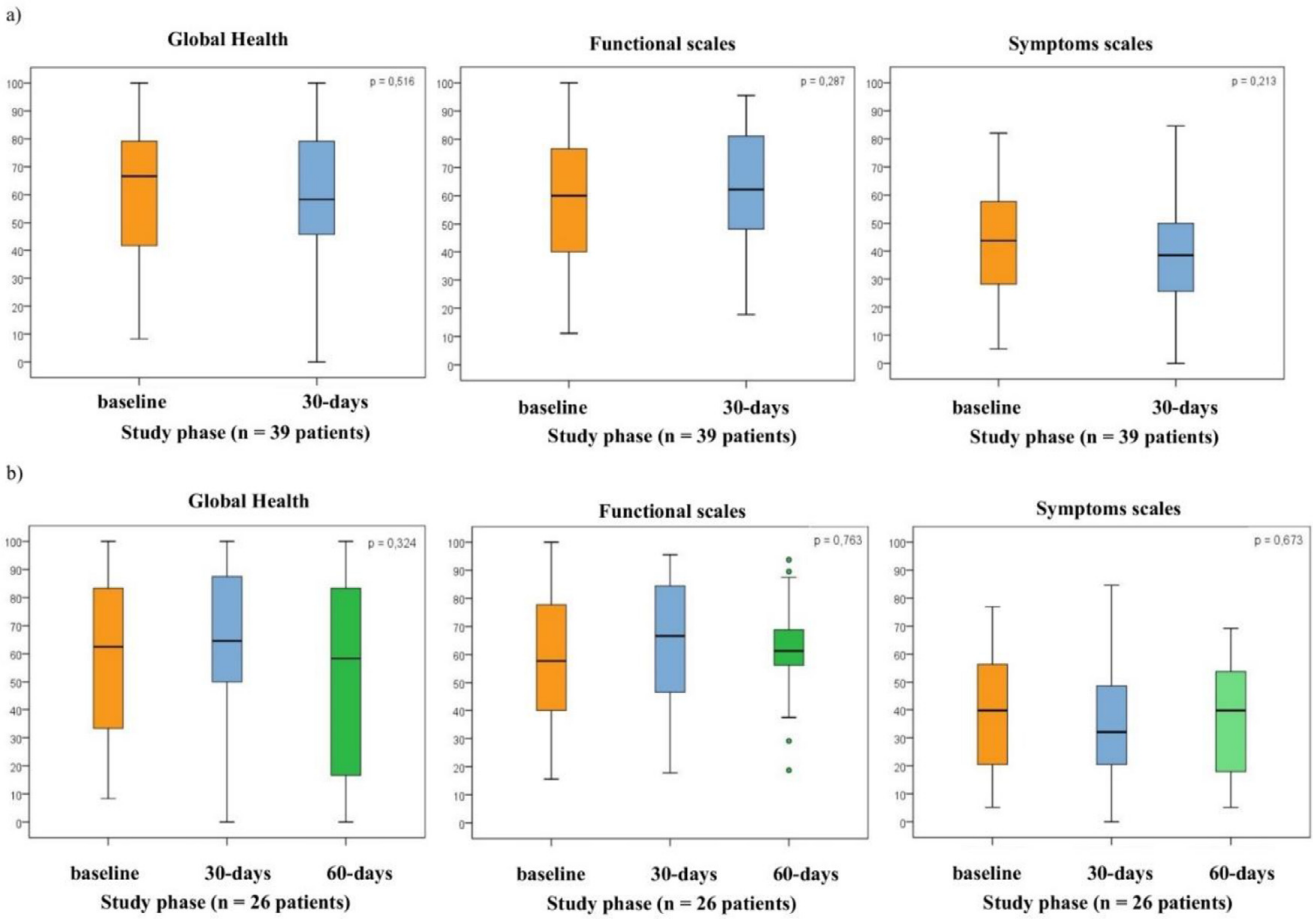
Box and whisker plots of EORTC QLQ-30 scores (in all domains; global health, functional scales, symptoms scales) at follow-up intervals. (a) Box and whisker plots of EORTC QLQ-30 score (in global health, functional scales, and symptoms scales) at initial and 30th day intervals. QoL scores did not change significantly at initial and throughout the 30th day in all domains. (*n* = 39 patients; p, Significance probability of the Student's *t*-test for paired samples). (b) Box and whisker plots of EORTC QLQ-30 score (in global health, functional scales, and symptoms scales) at initial, 30th day and 60th day intervals. QoL scores did not change significantly at initial and throughout the 30th day and 60th day, in all domains. (*n* = 26 patients); 27 cases without information (16 deaths + 11 cases without information); p, Significance probability of the Student's *t*-test for paired samples. The box shows the quartiles (the top and bottom of the boxes represents the 75th and 25th percentiles) of the domains in the QLQ-30 scores, while the whiskers extend to show the rest of distribution (the top and bottom of the whiskers represents the highest and lowest data points, excluding any outliers). The line within each box represents the median. The points are determined to be the outliers.

The authors had 56 participants at baseline, 46 on the 30th day, and 38 on the 60th day. Of these, 53 patients completed the QoL questionnaire at baseline, 39 on the 30th day, and 29 on the 60th day (Fig. 1). The initial mean scores for global health status, functional scale, symptoms scale, and QLQ-LC13 were as follows (mean ± SD): 59.6 ± 27, 54.8 ± 22.3, 44.7 ± 21.1, and 29.9 ± 11.9, respectively. The authors observed non-significant improvements in QoL between the baseline and the 30th day. Mean changes for global health status, functional scale, symptoms scale, and QLQ-LC13 were +3.5 (*p* = 0.516), +3.8 (*p* = 0.287), −4.7 (*p* = 0.213), and +1.8 (*p* = 0.423), respectively (Fig. 1a).

On the 30th and 60th days post-IPC, non-significant improvements were noted compared to the baseline: global health status: +4.8, −12.3 (*p* = 0.324); functional scale: −4.5, −1.9 (*p* = 0.763); symptom scale: −5.1, −2.2 (*p* = 0.673); and QLQ-LC13: −0.5, −1.9 (*p* = 0.780). Of the 27 patients excluded from this analysis, 16 were excluded because of death and 11 because of incomplete questionnaires at the end of the 60th day (Fig. 1b).

### Dyspnea and pain

**Fig. 2:**
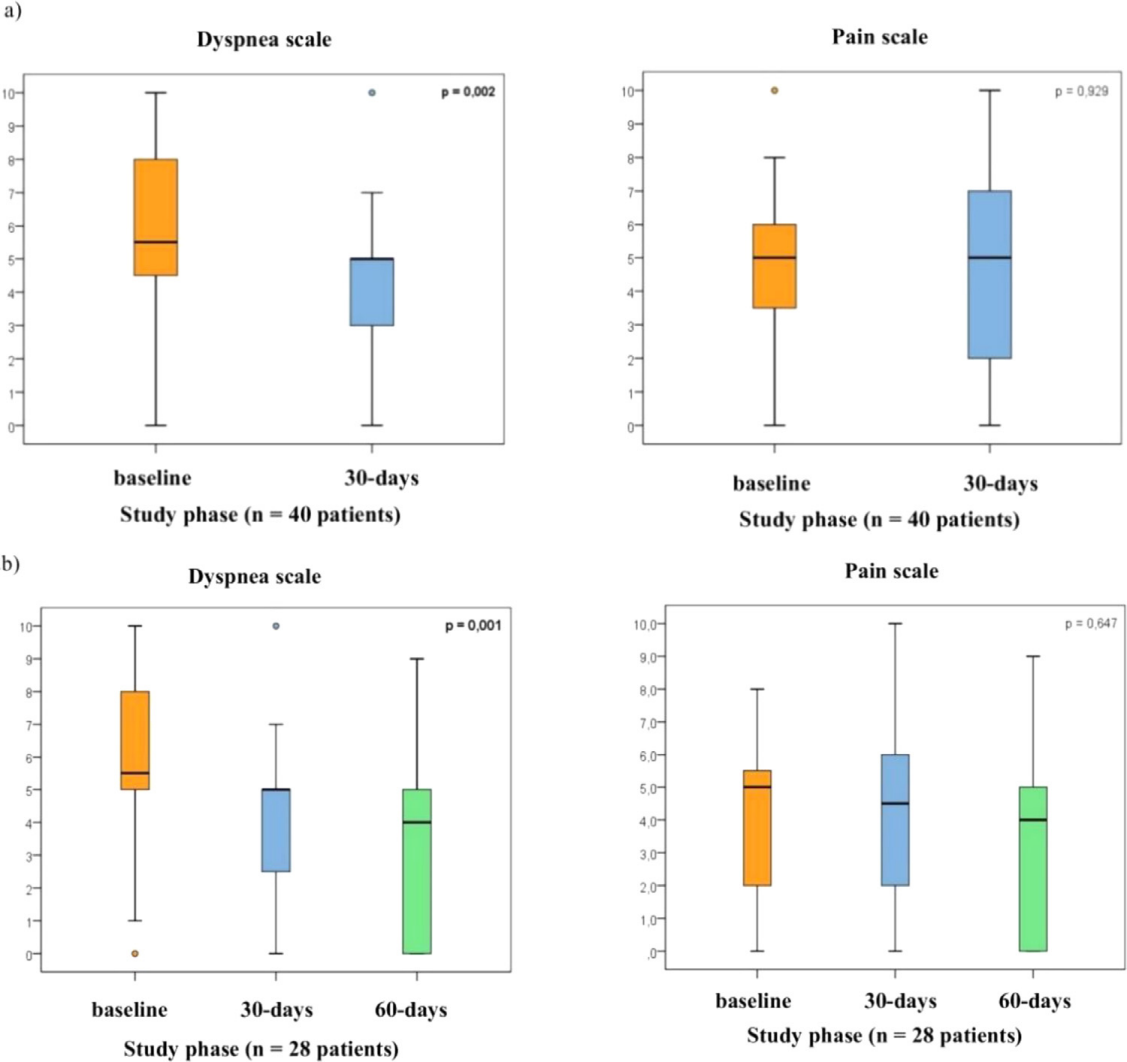
Box and whisker plots of VAS to assess dyspnea and pain at follow-up intervals. (a) Box and whisker plots of VAS to asses dyspnea and pain at initial and 30th day intervals (*n* = 40 patients; p, Significance probability of the Student's *t*-test for paired samples). (b) Box and whisker plots of VAS to asses dyspnea and pain at initial, 30th day and 60th day intervals. The analysis of the VAS showed a significant control of dyspnea at 30th day compared to initial, but pain did not change significantly. (*n* = 28 patients; p, Significance probability of the Student's *t*-test for paired samples). The box shows the quartiles (the top and bottom of the boxes represents the 75th and 25th percentiles) of VAS to asses dyspnea and pain, while the whiskers extend to show the rest of distribution (the top and bottom of the whiskers represents the highest and lowest data points, excluding any outliers). The line within each box represents the median. The points are determined to be the outliers.

In the inclusion phase, 53 patients completed the VAS dyspnea and pain scale. On the 30th day, the authors had 40, and on the 60th day, the authors had 28 completed questionnaires. Significant reductions in pain and dyspnea were noted with the VAS from baseline to the 7th day, with a mean change of −1.2 (*p* = 0.035), and −2.8 (*p* < 0.001), respectively. Improvements in dyspnea continued from the baseline to the 30th day, with a mean change of −1.6 (*p* = 0.002) (Fig. 2a).

At the end of the study, 28 patients completed the VAS and showed significant control in respiratory symptoms compared to the baseline, with a mean change of −2.5 (*p* = 0.001). No worsening of pain was noted compared to the baseline (mean change: −0.8; *p* = 0.647). Of the 25 patients excluded from this analysis, 16 were excluded because of death and 9 because of incomplete questionnaires (Fig. 2b).

### Survival

**Fig. 3:**
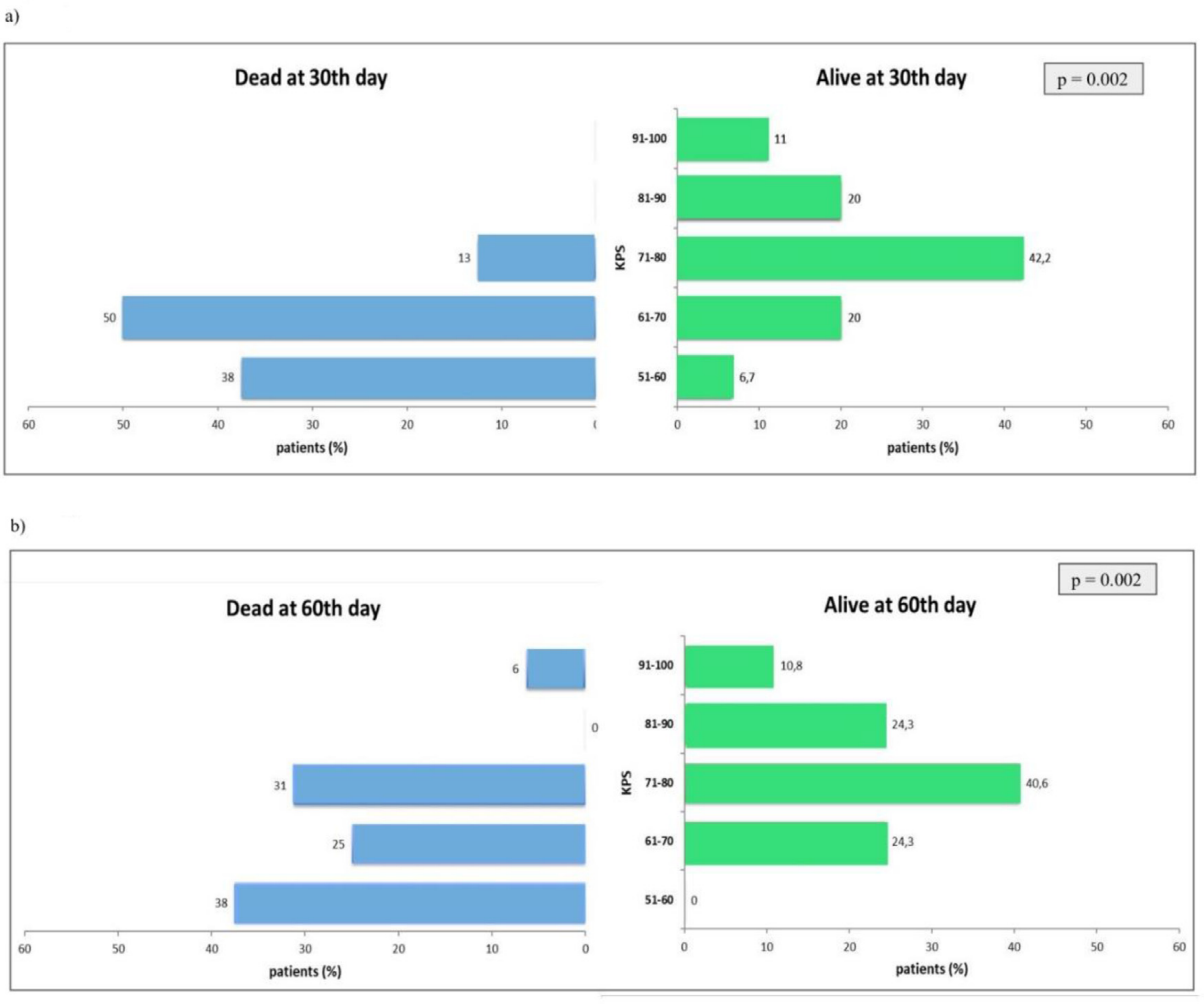
Comparison between those who died and those who survived during the first 30 and 60 days after IPC placement. The Fisher's exact test was used in the treatment of data regarding the association between initial performance status and mortality after 30th days of IPC placement. Comparison between those who died and those who survived during the first 30 (a) and 60 days (b) after IPC placement showed a significantly worse initial performance status (KPS).

At baseline, 53 patients completed the QoL questionnaires and the VAS scale, and 8 of them died during the first 30 days. An analysis of the mean scores at baseline compared patients who died and those who survived in the first 30 days. The initial average in the QLQ-13 score of patients who died was significantly worse than the group of living patients (mean ± SD: 35.1 ± 4.2 vs. 29.0 ± 12.6; *p* = 0.016). However, there were no significant changes in the QLQ-30 questionnaires. In contrast, patients who died were significantly more symptomatic than the corresponding group of living patients (dyspnea scores; mean ± SD: 7.8 ± 1.8 vs. 5.9 ± 2.4; *p* = 0.023). Similarly, patients who died until the 30th days and 60th days had a significantly worse initial performance status, as demonstrated by performance scores, compared to those who were alive at these two follow-up intervals (Fig. 3).

## Discussion

Patients with symptomatic MPE experienced significant relief from dyspnea without an increase in pain after catheter placement. Nevertheless, the QoL did not change significantly over time. Complication rates were acceptable and compared favorably with a large experience with IPC [12]. As expected, worse performance status and respiratory symptoms may indirectly influence mortality during the 30-day follow-up.

The most important disabling symptom caused by MPE is dyspnea, which has been reported in 50% of patients [2]. The present data showed rapid and persistent relief in dyspnea, measured by the VAS after IPC insertion on the 30th day, remaining statistically significant on the 60th day. A systematic review identified 12 studies in which 95.6% of patients reported improvement in dyspnea after IPC placement [13]. Putnam et al. used the dyspnea component to address QoL and reported an improvement in QoL at 30, 60 , and 90-days post-IPC [7].

Many studies have attempted to evaluate the impact of IPC on symptoms as a surrogate for QoL; however, most have not applied validated scoring tools in patients with malignancy [14,15]. The present study used validated and tested EORTC questionnaires to evaluate the QoL of an oncologic population before and after IPC placement [9]. The same tool was used in an Austrian study to assess the QoL of 85 patients with advanced cancer undergoing palliative treatment. The authors showed an expected worsening in functional scales and global health status/QoL during the patient's last 3-months of life [16]. The benefits of IPC have also been observed in other studies. In a Spanish cohort of 51 patients with recurrent MPE, IPC significantly improved EORTC QLQ symptom scores [17]. In contrast, the present study did not demonstrate any improvement or deterioration in the EORTC QoL 3 domains over time. Another study also related an overall improvement in QoL to the fact that IPCs can be inserted in an ambulatory setting, reducing hospitalization, and allowing self-management to achieve symptom control [5].

Complication rates were acceptable and compared favorably to those of the largest series of patients treated with IPC [12]. Empyema was likely to be among the most severe complications related to IPC and occurred in four patients, similar to the rates of 3.2% previously reported [12]. SP was observed in 45% of patients whose catheter was withdrawn, which is consistent with the work of Tremblay et al., with a rate of 43.9% in a total of 250 patients [12]. Since the introduction of IPC in the management of MPE, international studies in developed countries have reported its safety and efficacy 18, 19, 20. Nonetheless, to our knowledge, this is the largest study performed in a developing country to evaluate symptom control, QoL, and feasibility of IPC placement. The Brazilian public health system has some peculiarities that have not been seen in other countries. Despite being designed to be universal and serve the entire population, the private sector also plays an important role, with the public system being more accessible to the population of lower socioeconomic status. A study published in 2019 by the Brazilian Institute of Geography and Statistics (IBGE) showed that 71.5% of Brazilians depend solely on the Universal Health System (SUS) [21]. In addition, there are large regional differences, with some states having greater access to supplementary health services, such as São Paulo, where 38.4% of the population uses the private sector. This shows the impact of the present study by demonstrating the feasibility of using IPC in this population.

The cost of an IPC may appear as an issue because of the cost of the device and single-use vacuum bottles. The present study showed an average consumption of 1.7 bottles per patient per week, with a mean removal time of 47 days. In a randomized trial, Penz et al. compared the costs associated with the use of IPCs and talc pleurodesis [22]. The overall mean costs (SD) for managing patients with IPCs and talc pleurodesis were $4993 ($5529) and $4581 ($4359), respectively. IPC is significantly less costly than talc pleurodesis in patients with < 14 weeks of LE. Considering the short LE, the long-term need for vacuum bottles is unlikely; therefore, these results suggest that IPC might be a beneficial strategy for the public health system.

This study has several limitations, some of which are inherent to its design. First, this was a single-center study with small sample size, and the low compliance rates may have limited the strength of the conclusions. Given the nature of the disease, palliative care studies generally obtain high rates of missing data, and frequently, only a minority of patients complete all questionnaires. Although the compliance rates at different time points in the present study were low (68% at 30 days and 51% at 60 days), they did not differ considerably from those obtained in previous studies that examined similar cohorts [23,24]. There are several reasons why the patients did not complete the questionnaires. Some patients were lost to follow-up, while others experienced clinical deterioration and could not complete the questionnaires. However, the MORECare guidelines conclude that in palliative and end-of-life care, high attrition rates should not be considered indicative of poor design [25]. The absence of a control group also limits the analysis and quality of the results because QoL questionnaires often offer subjective results. Finally, the nature of the measured outcomes was subjective (VAS and QoL scores). The factor that may have allowed bias in self-reporting was that the treatment intent in MPE drainage was palliative. Therefore, a subjective, patient-reported measure is necessary and relevant.

## Conclusion

The present study demonstrated that IPC relieves respiratory symptoms without compromising QoL and has a low complication rate. It represents a suitable option for patients with MPE and short LE in the Brazilian public health system.

## Abbreviations

Malignant pleural effusion (MPE); Life expectancy (LE); Quality of life (QoL); Indwelling pleural catheter (IPC); European Organization for Research and Treatment of Cancer QoL (EORTC); Visual Analog Scale (VAS); Common Terminology Criteria for Adverse Events (CTCAE); Spontaneous pleurodesis (SP).

## Funding

This research did not receive any specific grant from funding agencies in the public, commercial, or not-for-profit sectors.

## Conflicts of interest

The authors declare no conflicts of interest.

## Appendix Supplementary materials

Image, application 1

Image, application 2
