# Supplementary material for: Quality of life in patients with malignant pleural effusion treated with an indwelling pleural catheter in an emerging country
Source: Clinics (Sao Paulo). 2022 Jun 18;77:100063. doi: 10.1016/j.clinsp.2022.100063 (PMC9214818; doi:10.1016/j.clinsp.2022.100063)
Supplement: Supplementary file 1 [file mmc1.pdf]

EORTC QLQ- LC13 Scoring Manual

The **Lung Cancer Module** is a supplementary questionnaire module to be employed in conjunction with the QLQ-C30. The QLQ-LC13 incorporates one multi-item scale to assess dyspnoea, and a series of single items assessing pain, coughing, sore mouth, dysphagia, peripheral neuropathy, alopecia, and haemoptysis.

The scoring approach for the QLQ-LC13 is identical in principle to that for the symptom scales / single-items of the QLQ-C30. All scoring information specific to the QLQ-LC13 is presented in Table 1.

Interpretation:
All of the scales and single-item measures range in score from 0 to 100. A high score for the scales and single items represents a high level of symptomatology or problems.

Table 1. Scoring the QLQ-LC13

	Scale	Number of items (n)	Item range*	QLQ-LC13 item numbers ($I_1, I_2, \ldots, I_n$)
**Symptom scales / items**				
Coughing	LCCO	1	3	31
Haemoptysis	LCHA	1	3	32
Dyspnoea [a]	LCDY	3[a]	3	33 - 35
Dyspnoea when resting [a]	*LCDYR*	*1*	*3*	*33*
Dyspnoea when walking [a]	*LCDYW*	*1*	*3*	*34*
Dyspnoea when stairs [a]	*LCDYS*	*1*	*3*	*35*
Sore mouth	LCSM	1	3	36
Dysphagia	LCDS	1	3	37
Peripheral neuropathy	LCPN	1	3	38
Alopecia	LCHR	1	3	39
Pain in chest	LCPC	1	3	40
Pain in arm or shoulder	LCPA	1	3	41
Pain in other parts	LCPO	1	3	42

* "Item range" is the difference between the possible maximum and the minimum response to individual items. All items are scored 1 to 4, giving range = 3.

[a] The dyspnoea scale should only be calculated if all three items have been answered. Some respondents ignore question 35 because they never climb stairs; in this case, the score for the dyspnoea scale would be biased if it were based upon the other two items. Hence if item 35 is missing then items 33 and 34 should be used as single-item measures.

Principle for scoring

1) **Raw score**

   For the multi-item scale, calculate the average of the corresponding items.

   $$Raw\ Score = RS = \left\{\frac{(I_1 + I_2 + \ldots + I_n)}{n}\right\}$$

   For each single-item measure, the score of the concerning item corresponds to the raw score.

   There are no reverse scoring items.

2) **Linear Transformation**

   To obtain the Score S, standardize the raw score to a 0 – 100 range using the following transformation:

   $$S = \left\{\frac{(RS-1)}{range}\right\} \times 100$$

For directions on Missing Data or for more detailed information on the Interpretation of Scores, we redirect to the EORTC QLQ-C30 Scoring Manual (2001).

Remark

The scoring of item 43 is optional.

Interpretation: A high score for item 43 represents a high level of pain relief.

Table 2. Scoring information for scoring item 43.

	Number of items	Item range	Item
Pain relief after medication*	1	3	43

* Item 43 might not be applicable and must only be scored if the answer to the question "Did you take any medication for pain?" is "Yes".

Reference

Bergman B, Aaronson NK, Ahmedzai S, Kaasa S, Sullivan M. The EORTC QLQ-LC13: a modular supplement to the EORTC Core Quality of Life Questionnaire (QLQ-C30) for use in lung cancer clinical trials. EORTC Study Group on Quality of Life. Eur J Cancer. 1994;30A(5):635-42.

Further questions or remarks regarding the scoring algorithms for the QLQ-LC13 can be directed to the QOL Specialist at the Quality of Life Department of the EORTC.
